# Polymers in Carbon Dots: A Review

**DOI:** 10.3390/polym9020067

**Published:** 2017-02-16

**Authors:** Yiqun Zhou, Shiv K. Sharma, Zhili Peng, Roger M. Leblanc

**Affiliations:** Department of Chemistry, University of Miami, Coral Gables, FL 33146, USA; yxz431@miami.edu (Y.Z.); sks38@miami.edu (S.K.S.); z.peng@umiami.edu (Z.P.)

**Keywords:** carbon dots, polymers, composites, polymerization, surface modification

## Abstract

Carbon dots (CDs) have been widely studied since their discovery in 2004 as a green substitute of the traditional quantum dots due to their excellent photoluminescence (PL) and high biocompatibility. Meanwhile, polymers have increasingly become an important component for both synthesis and modification of CDs to provide polymeric matrix and enhance their PL property. Furthermore, critical analysis of composites of CDs and polymers has not been available. Herein, in this review, we summarized the use of polymers in the synthesis and functionalization of CDs, and the applications of these CDs in various fields.

## 1. Introduction

Nanotechnology has become a popular topic since the start of the 21st century in a variety of fields including: drug synthesis and delivery [1], environmental protection [2], electronics manufacture [3], and astronomy [4], all of which can be attributed to the nano scale particles and their unique properties endowed by their nano scale size. Carbon dots (CDs) are based on the prior quantum dots (QDs) which had a promising background, as they exhibited excellent optical properties. However, due to the heavy metal elements such as Cd [5] involved in the QDs structure, attention has been switched to its green substitutes. Meanwhile, CDs were discovered when researchers tried to purify single-walled carbon nanotubes through preparative electrophoresis in 2004 [6]. Since then, numerous research projects have been developed on the synthetic approach of CDs including chemical ablation [7,8], electrochemical carbonization [9], laser ablation [10], microwave irradiation [11] and hydrothermal/solvothermal treatment [12,13,14]. Various characterization methods including UV/Vis, fluorescence, FTIR, Mass, NMR, atomic-force microscopy (AFM), transmission electron microscopy (TEM), scanning electron microscopy (SEM) and X-ray photoelectron spectroscopy (XPS) have been used for the analysis of CDs. According to these studies, it is generally concluded that CDs are a new type of fluorescent carbon based nanoparticles (1~10 nm) whose spectroscopic characteristics can be described as follows.

(1) There are two characteristic peaks in their UV/Vis spectra which correspond to aromatic C=C π-π* transition (~263 nm) [13,15,16] and C=O n-π* transition (~332 nm) [16,17,18], respectively.
(2) CDs are photoluminescent, which indicates CDs (especially the surface energy traps) after absorption of photons will generate excitons which will quickly recombine to release the energy in the form of light emission. CDs are commonly excitation wavelength dependent shown in various fluorescence spectra [18,19,20,21]. As for the mechanism of the photoluminescence (PL) of CDs, there exist two prevalent explanations from the viewpoints of surface state in CDs and quantum size effect, respectively [22].
(3) From FTIR spectra, the functional groups of CDs include –OH, –COOH, –C=C– [23,24], and also –NH_2_ and –CONH– depending on the preparation methods and raw materials [25,26].
(4) Based on XPS measurement, CDs basically consist of C, O [27] and other elements depending on raw materials as well [16,26,28].

The most important property of CDs is their PL and this feature is determined by their intrinsic nanostructure. Therefore, to enhance the PL properties of CDs, attention should be turned to the initial synthesis to control their structure. To this end, polymers have become preferred carbon precursors [29,30] for the preparation of CDs due to their known macromolecular structure, which would provide templates for synthesizing CDs with clear structure. This is quite significant for those who have made efforts to figure out the structure of CDs as well as their PL mechanism. In addition, surface passivation and functionalization are among the most effective ways to enhance the PL property of CDs by modifying their surficial structure. Polymers such as PEG have been widely used in both passivation [31,32] and functionalization process [33,34,35].

There are many review papers on polymers and CDs but very little work has been done in assimilating the CDs incorporated to the polymers. In this review paper, we describe the recent advances in CDs which are embedded in polymeric gel [36,37], molecularly imprinting polymers [38,39], and polymer composite films [40,41], and compare the efforts made so far to improve the PL. The polymer composites provide the polymeric matrix for CDs while the special PL properties of CDs make polymers more applicable in fields such as bioimaging, biosensing and drug delivery. Therefore, polymers have gradually widely permeated in every aspect of CDs.

## 2. Polymers as Precursors for CDs Preparation

### 2.1. Polymers as Direct Precursors for CDs Synthesis

Owing to their natural macromolecular structures, polymers are excellent candidates for the synthesis of CDs, providing matrix and template. Therefore, polymers or polymeric chemicals have gradually drawn increasing interest to be used as carbon source for the synthesis of CDs. In respect of the nature of the polymers used for synthesizing CDs, both natural polymers, such as lignin and chitosan [42,43,44,45], as well as synthetic polymers, such as polyethylene glycol (PEG) [46,47,48], have been reported.

**Figure 1:**
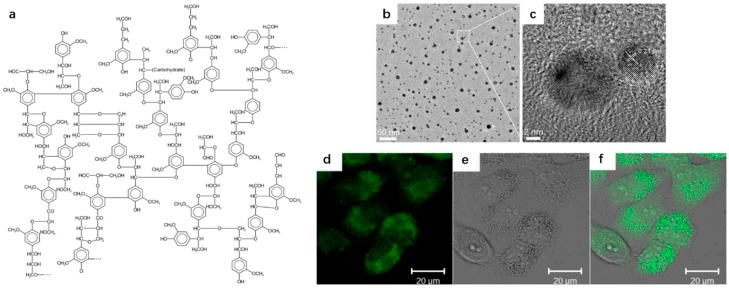
(**a**) The molecular structure of lignin; (**b**) TEM and (**c**) HRTEM images of CDs; (**d**) a confocal fluorescence microphotograph of Hela cells labeled with the CDs (λ ex: 405 nm); (**e**) a bright field microphotograph of the cells; and (**f**) an overlay image of (**d**,**e**). Figure adapted from Ref. [42] with permissions from the publishers.

As for natural polymers as precursors, Chen et al. developed a rapid approach to produce CDs by hydrothermal treatment (pyrolysis) of lignin in the presence of H_2_O_2_ [42]. Lignin (Figure 1a) being an abundant natural cross-link phenolic polymer and an excellent source of carbon and H_2_O_2_ being one of the strongest oxidizing agents known in acidic condition (1.8 eV, oxidation potential); the latter can split into hydroxyl radicals (•OH), which are highly active, under the photo-assisted catalysis Fe^3+^/Fe^2+^ in water [49]. The resulting •OH can break the strong carbon–carbon linkage of lignin to form CDs. TEM and high-resolution TEM (HRTEM) were performed on the resulting CDs and the results (shown in Figure 1b,c) indicated the size of CDs ranged from 2 to 10 nm. In addition, from HRTEM image, it was also observed that the CDs owned crystalline structure and the lattice spacing distance was measured to be 0.21 nm. The resulting CDs have been introduced into the Hela cells and the PL spots have been observed in the cell membranes and cytoplasmic area, which indicate that CDs can penetrate the Hela cell. The confocal fluorescence microphotograph of the Hela cells labeled with CDs are demonstrated in Figure 1d–f.

Chitosan is a linear polysaccharide that can be easily acquired from the shells of shrimp and other sea crustaceans for commercial purposes [50]. Majumdar and coworkers synthesized fluorescent CDs by heating a chitosan hydrogel in a microwave [29]. It is claimed as the first report of synthesizing CDs by using chitosan gel as precursors. The chitosan gel is stable without degradation and at the same time it will increase the shelf life of starting materials to obtain CDs. Besides, it was also found that the PL properties of such CDs were affected by different pH conditions. For instance, when CDs were prepared at pH 3, the PL showed a sharp peak while at pH 1 or 5, they exhibited a broad emission peak in the PL spectra.

In addition, other natural polymeric products such as cellulose and sugarcane bagasse pulp have also been developed to synthesize highly photoluminescent CDs. Da Silva Souza et al. demonstrated CDs could be prepared from cellulose nanocrystals using different pyrolysis temperatures along with the discovery of different carbon structures determined by different cellulose nanocrystals arrangement [51]. The prepared CDs were composed of both graphitic and amorphous carbon with a size distributed between 4 and 8 nm. In addition, the PL covers the blue-to-green region. Shankaran et al. also mentioned a green synthesis of CDs using sugarcane pulp as the renewable carbon precursor [52]. The obtained CDs exhibited relatively high PL with a QY of 18.7%, and HRTEM and AFM indicated the CDs owned the size of around 4.1 nm and surface thickness of 5 nm. Besides, the CDs were highly crystalline with face centered cubic crystal structure by XRD and TEM analyses, which showed great prospect in the future application in drug-delivery.

**Figure 2:**
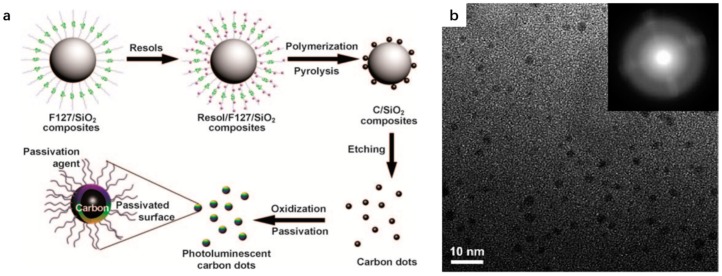
(**a**) Processing diagram for the synthesis of photoluminescent CDs; and (**b**) HRTEM image of CDs passivated with PEG_1500N_. The inset is the SAED pattern. Figure adapted from Ref. [48] with permissions from the publishers.

In terms of synthetic polymer precursors, resoles (phenol/formaldehyde resins, *M*_w_ < 500) have been reported by Liu et al. as carbon source to build up a type of CDs with amorphous structure by using surfactant-modified silica nanospheres as carriers [48]. The overall synthetic procedure is illustrated in Figure 2a. Here, silica colloid spheres were functionalized with amphiphilic triblock copolymer F127 (EO_106_PO_70_EO_106_, Mol. Wt. = 12,600; EO = ethylene oxide, PO = propylene oxide) to synthesize satellite-like F127/SiO_2_ composites, surfactant-modified silica nanospheres. Then resoles were further polymerized on the surface of silica nanospheres. The subsequent pyrolysis and etching assisted the formation of CDs and removal of carriers, respectively. The main point of the method is the silica nanosphere carriers do not only provide anchors for the polymerization of resoles but also inhibit the aggregation of CDs during pyrolysis. HRTEM spectroscopy was performed on the obtained CDs and the experimental result (Figure 2b) revealed the size of CDs was narrowly distributed in the range 1.5–2.5 nm. Besides, the lack of lattice structures and the diffuse ring pattern obtained by selected area electron diffraction (SAED; inset in Figure 2b) suggested the CDs are amorphous.

PEGs are another type of synthesized polymers that have been widely used as the precursors for the preparation of CDs. For example, Li et al. reported a low-cost electrolysis method to obtain high-PL CDs with PEG series compounds as the carbon source [53]. The resulting CDs were great electron donors/acceptors and had higher proton absorption ability than any other CDs due to their structure. Furthermore, in comparison, PEG_200_, PEG_600_ and PEG_800_ were employed to achieve high PL, among which CDs using PEG_600_ as the carbon precursor exhibited the highest PL intensity with a quantum yield (QY) of 38%. Due to their high PL intensity, the CDs were conjugated with tyrosinase to serve as a fast, sensitive, and accurate biosensor to detect L-DOPA with a detection limit of 9.0 × 10^−8^ mol·L^−1^.

**Figure 3:**
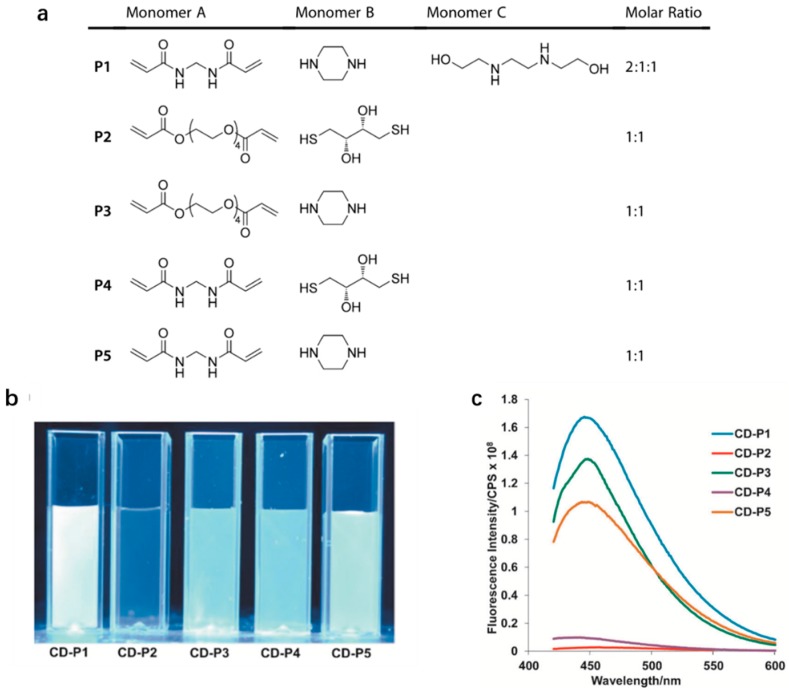
(**a**) Monomers and the molar ratios used in the synthesis of P1–P5; (**b**) aqueous solutions of CDs CD-P1 to CD-P5 (0.1% *w*/*v*) excited at 365 nm; and (**c**) PL spectra of CD-P1 to CD-P5 (λ ex 400 nm). Figure adapted from Ref. [54] with permissions from the publishers.

Jiang et al. performed synthesis work by a thermopyrolysis of five types of 1,4-addition polymers (P1–P5) which were selectively constructed from monomers including piperazine, dithiothreitol (DTT), *N*,*N*′-bis(2-hydroxyethyl) ethylenediamine (BHEEDA), *N*,*N*′-methylenebisacrylamide (MBA) and tetra (ethylene glycol) diacrylate (TEGDA) [54]. The specific monomers with certain molar ratios to synthesize each 1,4-addition polymers are shown in Figure 3a. In Figure 3a, we can observe all monomers A are α,β-unsaturated carbonyl compounds which are attacked by nucleophiles (monomers B and C) at the β carbon to occur 1,4-addition. Eventually, these 1,4-addition polymers theoretically provided templates to define the structures and properties of CDs, which was of great benefits to figure out the PL mechanism of CDs. Then thermopyrolysis of P1–P5 was carried out at 250 °C for 2 h to obtain five types of CDs (CD-P1, CD-P2, CD-P3, CD-P4 and CD-P5) (Figure 3b). These five types of CDs were prepared as aqueous solution with the same concentration and were excited at 400 nm. The result was shown in Figure 3c. Considering final QY, CDs made from P1 owns the highest QY (26%) and the PL results showed that the PL of nitrogen containing CDs were higher than that of those not containing nitrogen.

**Figure 4:**
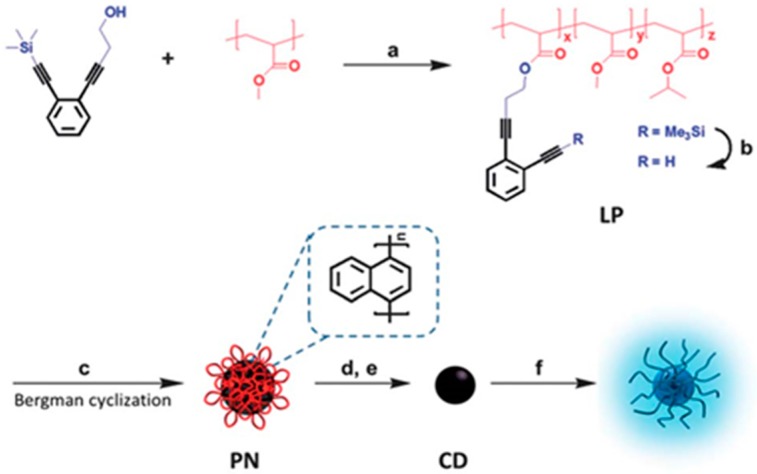
Illustration of the preparation of photoluminescent CDs: (**a**) indicates the synthesis of P(MA-r-EDY); (**b**) means deprotection of trimethylsilyl groups; (**c**) shows the formation of polymeric nanoparticles; (**d**) indicates the carbonization of the polymeric nanoparticles; (**e**) means the formation of CDs with passivated surface state; and (**f**) demonstrates functionalization of CDs surface. Figure adapted from Ref. [47] with permissions from the publishers.

As a hard question to answer, PL mechanism is still under debate. Therefore, to clarify the PL mechanism of CDs, a novel bijective approach has been employed by Zhu et al. to fabricate CDs (17%, QY) from size-tunable single chain polymeric nanoparticles obtained by mixing diphenyl ether, PMA-EDY (EDY indicates 4-((2-(2-Trimethylsilyl)ethynyl)-phenyl)but-3-yn-1-ol) and ethyle acetate [47]. Figure 4 shows the detailed synthesis process of the CDs. Owing to the living free radical polymerization technique and Bergman cyclization, narrowly dispersed CDs were acquired and PL was explained by the consequence of electron-hole pair recombination in localized sp^2^ carbon clusters, which was generally considered as the luminescent center. Besides, CDs were divided into two classes viz with graphitized carbon core and with disordered carbon core, based on the presence of predominantly sp^2^ carbon in the core of CDs.

### 2.2. Polymers as Indirect Precursors for CDs Synthesis

Besides, as most reports indicate, the formation of CDs needs to undergo polymerization and carbonization [55,56,57,58], which means most CDs own polymeric structures among which poly aromatic structure has often been mentioned [56,59,60,61] relying on UV/Vis absorption spectra as well as XPS data. Here, we summarize some compounds that have been mentioned to experience polymerization process to produce CDs. Since different functional groups own different energy traps, which results in the various PL behaviors of CDs [22], it is quite important to select proper monomers to participate in the formation of CDs.

**Figure 5:**
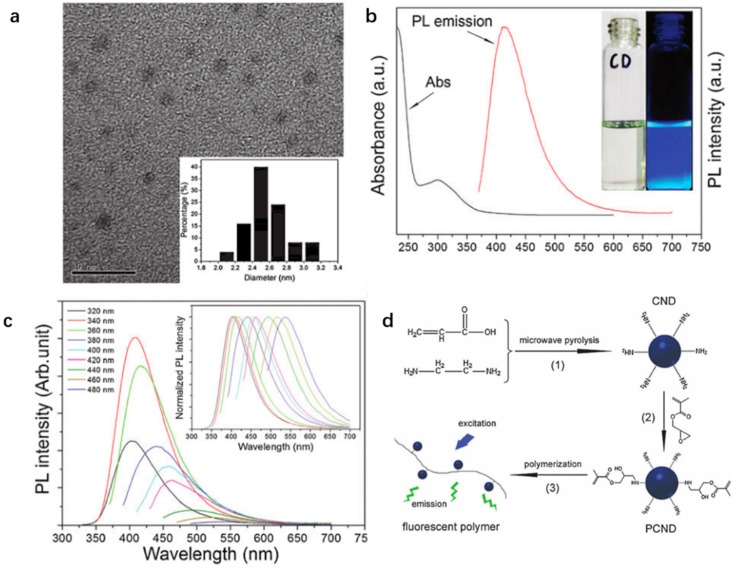
(**a**) HRTEM image of CNDs (scale bar: 10 nm) with a narrow size distribution of 2.0–3.2 nm in diameter; (**b**) UV/Vis spectrum (λ_ab_ = 300 nm) and PL emission spectrum (λ_ex_ = 360 nm) of an aqueous solution of the CNDs (1 mg·mL^−1^) with an emission peak at 420 nm; (**c**) PL emission spectra of the CNDs aqueous solution under excitation with different wavelengths (inset is the normalized PL emission spectra); and (**d**) synthesis procedure of: CNDs (1); PCNDs (2); and fluorescent polymers (3). Figure adapted from Ref. [57] with permissions from the publishers.

Zhang and coworkers used acrylic acid and 1,2-ethylenediamine (EDA) (1:1, molar ratio) as carbon source and passivation agent, respectively, to synthesize CDs (CNDs) [57] (Figure 5d). Since the CDs are enriched with amine surface groups, it allows further functionalization with glycidyl methacrylate (GMA) to produce polymerizable CDs (PCNDs) which is followed by the polymerization reaction to achieve fluorescent polymers. The authors claim it as the first ever report for the combination of CDs with other representative acrylamide and methacrylate type monomers to synthesize fluorescent polymers. The HRTEM image (Figure 5a) reveals the size of the CNDs is narrowly distributed within 2.0–3.2 nm in diameter which is close to that measured of CDs synthesized with polymers as direct precursors [44], revealing the uniformity of the size of CDs. Besides, the UV/Vis absorption and PL emission spectra (Figure 5b) are consistent with that characterized of most CDs [43]. However, the PL emission spectra under excitation with different wavelengths (Figure 5c) show a wide spectral width of PL of more than 350 nm which greatly broadens their application in bioimaging.

**Figure 6:**
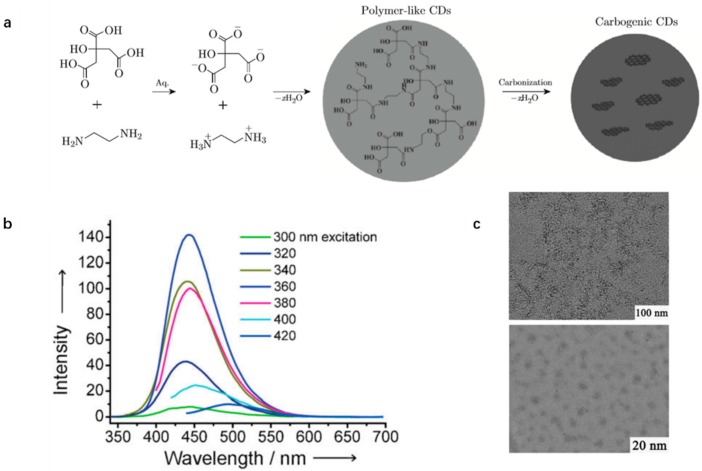
(**a**) Reaction mechanism of CDs synthesized by CA and EDA; (**b**) excitation-dependent PL spectra of CDs; and (**c**) TEM (upper) and HRTEM (lower) images of CDs. Figure adapted from Ref. [61] with permissions from the publishers.

Zhu et al. obtained high photoluminescent CDs with a hydrothermal method with citric acid (CA) and EDA as the precursors and passivation agent, respectively [61]. The resulting CDs exhibited the highest QY (80%) up to now and were even comparable to fluorescent dyes. The high QY was analyzed by using a femtosecond broadband (350–800 nm) transient absorption (TA) spectroscopy excited at 400 nm. From the TA spectra, the decay is rather little in all wavelengths indicating the high stable excited-state of CDs which contributes to the high QY. CA and amine compounds due to their populated functional groups and flexible binding pattern have become classic constructive carbon precursors and passivation agent for the synthesis of CDs (Table 1). However, the mechanism (Figure 6a) and structure of such prepared CDs are more complicated than other CDs because of unclear bond patterns. In addition, the obtained CDs, though own high PL QY, own relatively narrow spectral width (250 nm) of PL when the size of CDs is aligned to be 2–6 nm shown in TEM (upper, Figure 6c) while the HRTEM image (lower, Figure 6c) reveals most CDs nanoparticles are amorphous without any lattices.

**Figure 7:**
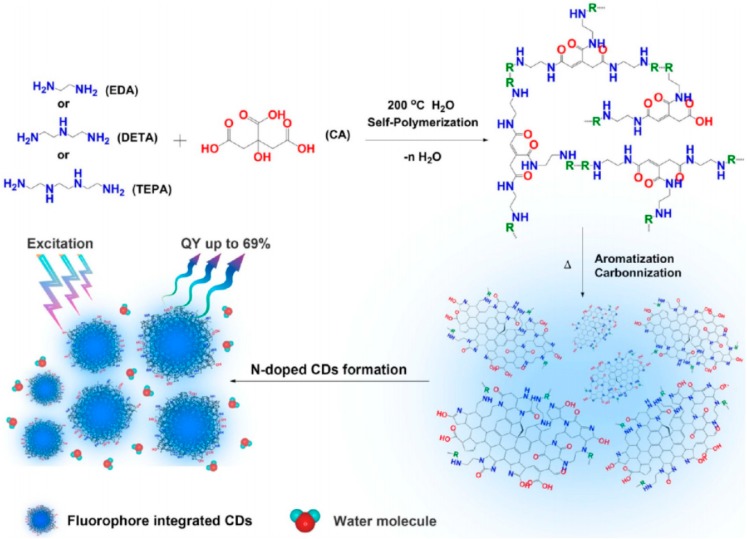
Proposed formation pathway of N-doped CDs. Figure adapted from Ref. [56] with permissions from the publishers.

Besides, Wang et al. synthesized photoluminescent CDs by hydrothermal reaction of CA and three different linear polyethylenic amine (PEA) molecules i.e., EDA, diethylenetriamine (DETA), and triethylenetetramine (TEPA) with molar ratio of 2:1 (Figure 7) to investigate the structure effect on the enhanced PL of CDs [56]. The QY was calculated as 69.3%, 68% and 33.4%, and the PL lifetime (τ) was measured as 14, 13 and 10 ns for CD-EDA_(2/1)_, CDs-DETA_(2/1)_ and CDs-TEPA_(2/1)_, respectively. The experimental results imply that either the structures or the molar ratio of PEA will influence the PL properties of these CDs, and the hybridization between the surface state and carbon backbone affected the chemical or physical properties of these CDs, for instance, the increasing of amine groups in the PEA molecules can also govern the molecular state of the CDs formation. Besides, the content of conjugated π-domains with C=N in the carbon backbone is in relation to their PL QY (up to 69%). In addition, the PL stabilities tests of CD-EDA_(2/1)_, CDs-DETA_(2/1)_ and CDs-TEPA_(2/1)_ show the longer-chain PEA molecules is beneficial to keep long stability of CDs while shorter-chain PEA molecules, though own high QY, exhibit strong photobleaching behaviors similar to organic fluorophores.

## 3. Polymers for Surface Modification of CDs

Bare surface of CDs is rather defenseless due to the existence of many defect sites, such as dangling bonds, non-radiative states and radicals [71], which usually cause the low PL efficiency (QY) and impair of the optoelectronic properties of CDs. Surface passivation is the most effective technique to enhance the PL intensity and common polymer passivation agents include polyethylene glycol (PEG) [72,73], polyethyleneimine (PEI) [73,74], poly(ethylenimide)-*co*-poly(ethylene glycol)-*co*-poly(ethyl-enimide) (PPEI) [72] and 4,7,10-trioxa-1,13-tridecanediamine (TTDDA) [73,75]. The mechanism of surface passivation can be explained by cutting off other non-emitting ways to amplify the emission intensity caused by irradiation. After passivation with polymers or other organic molecules, the QY of CDs can increase dramatically [76].

Besides, functionalization usually occurs along with the passivation process and they are the same process when polymer passivation agents are doped. Functionalization is the process of adding new features and properties to a material by changing the surface chemistry of the material and is a useful technique to develop more tailoring work for CDs. After passivation, it can also modify the surface of CDs by bringing in or generating various functional groups such as carboxyl, carbonyl, and hydroxyl groups in the defect sites of their surface. The process is usually assisted by doping polymers or organic compounds. In terms of the category of doping, there are currently N-doping [28,77,78], S-doping [79,80], P-doping [81,82], and Si-doping [83,84], among which N-doping has proved to be the most effective and popular doping method.

Sun et al. in 2006 initiated diamine-terminated oligomeric poly (ethylene glycol) H_2_NCH_2_(CH_2_CH_2_O)_n_CH_2_CH_2_CH_2_NH_2_ (average n ~ 35, PEG_1500N_) as surface passivation agent [72]. When CDs were excited at 400 nm, the observed QY ranged from 4% to over 10% which varied due to the extent of surface passivation. The QY of CDs was comparable with those of traditionally prepared silicon nanocrystals after surface passivation. Mechanistically, the PL of CDs was attributed to the surface energy traps, which were caused by plenty of functional groups such as –NH_2_ on the surface of CDs that emitted light upon stabilization because of the surface passivation.

Wang et al. prepared CDs sample with an oligomeric PEG diamine (PEG_1500N_) as passivation agent [85], which was based on other previous reports [86] and later simply fractionalized CDs on an aqueous gel column. After separation, the most fluorescent fraction even achieved QY close to 60%, comparable to those of the best commercial CdSe/ZnS QDs in solution and brighter at the individual dot level. Furthermore, Goncalves and his coworkers synthesized CDs by laser ablation [34]. Then the CDs were capped with PEG_200_ and mercaptosuccinic acid. The CDs obtained from laser ablation were not fluorescent and the functionalization process with PEG_200_ endowed the CDs with PL.

In 2014, Liu et al. reported conjugating CA with a multifunctional ribonuclease A (RNase A) to prepare ribonuclease A-conjugated C-dot nanoclusters (RNase A@C-dots) for synchronous cancer imaging and therapy [31]. In the experiment, except the CDs obtained by conjugating CA and RNase A, in control experiment, CA without RNase was treated in the same condition and followed by the addition of PEG_2000N_ (mass ration, CDs/PEG_2000N_ = 1:20) to obtain C-dots-PEG_2000N_. It turned out that the PL of C-dots-PEG_2000N_ (4.33%) is five times of that of the bare CDs (0.87%), which exhibited the eminent enhancement of PL properties of CDs by surface passivation of PEG series.

**Figure 8:**
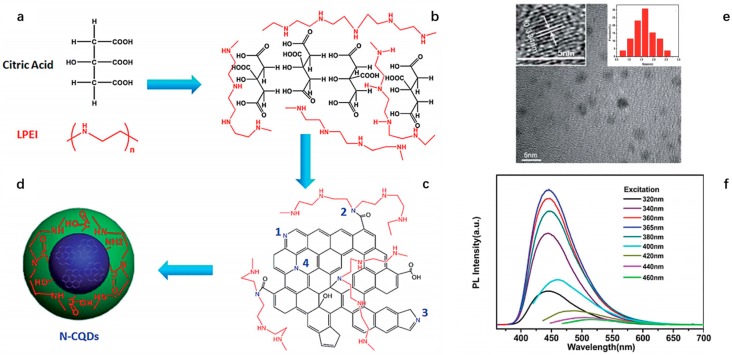
Proposed formation pathway, composition, and structures of as-obtained N-CDs: (**a**) polymerization of final N-CDs; (**b**,**c**) incorporation of N atom to final N-CDs (1, 3, and 4 indicate N atom existing in the aromatic framework and 2 shows LPEI linked to N-CDs by formation of amide groups); (**d**) surface passivation of N-CDs by –COOH, –OH, amine groups and LPEI chains; (**e**) TEM image of N-CDs (inset, HRTEM image and size distribution of 1–3 nm in diameter with 1.67 nm on average); and (**f**) PL spectra under excitation wavelength shorter than 500 nm. Figure adapted from Ref. [58] with permissions from the publishers.

Except PEG series compounds, other polyamine compounds have also been used as passivation agent in the synthesis of CDs [61,63,69]. Liu et al. prepared nitrogen-doped CDs (N-CDs) by using CA and linear-structured polyethyleneimine (LPEI) polymer as initial materials [58]. The LPEI serves not only as a nitrogen source but also as a surface-passivation agent for surface modification. The QY of the resulting CDs is 37.4%. The proposed structural mechanism is intermolecular and/or intramolecular dehydration which occurs among the –COOH, –OH groups and –H in the CA and further polymerization, aromatization and carbonization processes follow to form aromatic sp^2^ carbon clusters [61,87]. At the same time, the LPEI with rich amine groups can also react with CA (e.g., formation of amide N–C=O), thus incorporating N atom into the final nitrogen-doped CDs (Figure 8a–d). The TEM and HRTEM images (Figure 8e) of the obtained N-CDs revealed the average diameter of CDs is about 1.67 nm with high crystallinity with a lattice spacing distance of 0.334 nm, which is comparable with that of (002) plane of graphitic carbon. In addition, the PL spectra of N-CDs (Figure 8f) suggest the N-CDs have a wide spectral width (over 250 nm) of PL over which N-CDs exhibit strong PL.

**Figure 9:**
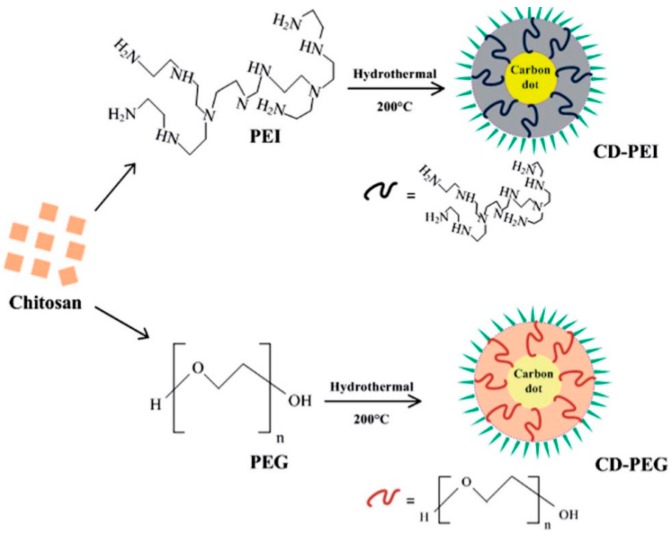
Schematic diagram depicting one-pot hydrothermal synthesis of CD-PEI and CD-PEG. Figure adapted from Ref. [88] with permissions from the publishers.

Gopinath and his colleagues prepared CDs using chitosan as starting carbon source and passivate the CDs by PEI and PEG (Figure 9), separately [88]. The QY of CD-PEI and CD-PEG was measured using quinine sulfate as a standard and found to be 13.15% and 7.01%, respectively, which was adequately bright for bioimaging as well as higher than their earlier report values [33]. In addition, the mean PL decay lifetime of CD-PEI and CD-PEG was 6.193 and 4.825 ns, respectively. Even though the carbon source remained the same, PL properties of CD-PEI were amazingly good compared to CD-PEG in terms of higher QY and longer PL decay lifetimes. Besides, fluorescence microscopic and spectroscopic analysis predict CD-PEI as a superior bioimaging agent compared to CD-PEG, owing to its efficient fluorescent characteristics and tunable emission from blue to red under cell culture conditions. Therefore, the surface of CDs, selection of right polymer groups for surface passivation can enhance its bioimaging efficiency.

**Figure 10:**
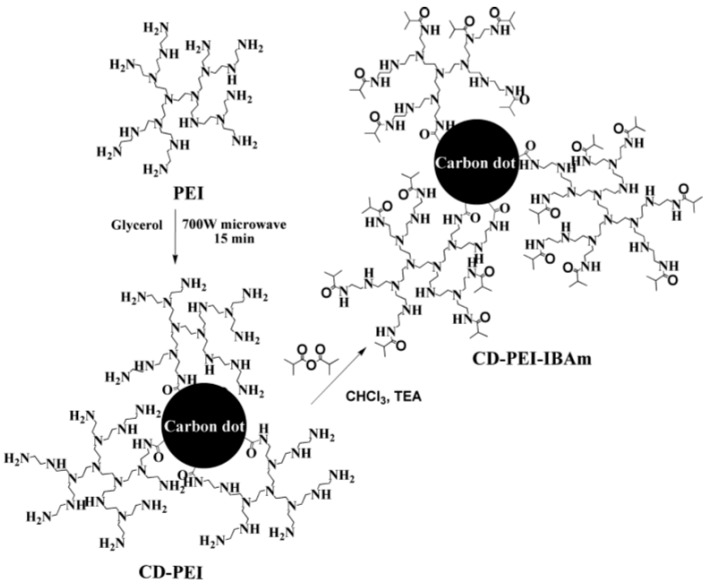
Preparation of Multi Stimuli-Responsive CDs. Figure adapted from Ref. [89] with permissions from the publishers.

Yin et al. integrated thermoresponsive hyperbranched PEI with multistimuli-responsive CDs [89] (Figure 10), which results in photoluminescent CD-PEI. After the amidation reaction of isobutyric anhydride and the PEI moiety, isobutyric amide (IBAm) groups were attached to CD-PEI to fabricate CD-PEI-IBAms. The CD-PEI-IBAms were also photoluminescent and their PL was barely affected by pH, salts, and the organic guests encapsulated while the cloud point temperature (T_cp_) of aqueous solution of CD-PEI-IBAms could be modulated by pH, salts and the organic guests encapsulated. Above all, CD-PEI-IBAms integrate the properties or functions such as multistimuli-response, nanocarrier for organic guest, and PL, which endows them as promising “smart” materials in the future application of biomedicine and biotechnology.

## 4. Polymer in Polymer–CDs Composite

### 4.1. CDs-Polymeric Gel Conjugation

When a three-dimensional polymeric network is loosely crosslinked, a hydrogel is formed [90]. They usually get swollen in water but do not dissolve in water. There have been many studies in the incorporation of nanomaterials in gel [91,92,93].

Gopinath and co-workers have made an intensive study on the incorporation of carbon dots in polymeric hydrogels loaded with an anticancer drug, 5-Fluorouracil (5-FU@CD-HY) [37]. In this work, they studied the multifunctional aspects of 5-FU@CD-HY using A549 (lung cancer) cell line as an in vitro model system and have found that 5-FU@CD-HY owned high PL, large surface area, strong mechanical strength and pH dependent behavior. All characteristics made 5-FU@CD-HY as a nanotheranostic system to monitor the cellular uptake and cytotoxic effects of anticancer drugs.

In another study, ion sensing applications of carbon dot–hydrogel hybrid nanomaterials using low molecular weight gelators (LMWGs) designed to provide a hydrophobic environment suitable for fluorescent ion sensing within an aqueous medium has been discussed [94]. It has been reported that the carbon dots showed an enhanced gelation properties and the hydrogel resulted an increased PL which was later employed as a useful sensor to detect heavy metals like Pb^2+^. Further work for colorimetric-optical sensing of heavy metals like Cr^6+^, Cu^2+^, Fe^3+^, Pb^2+^, Mn^2+^ by the use of carbon dots incorporated hydrogel has been reported by Gogoi et al. [95]. They have introduced chitosan based carbon dots rooted agarose hydrogel film as a hybrid solid sensing platform. It has been observed that the minimum detection limit was 1 pM for Cr^6+^, 0.5 nM for Fe^3+^, Pb^2+^, and Mn^2+^, and 0.5 μM for Cu^2+^. Beside metal ions, Baruah et al. have shown that Fluorescent carbon dots (CDs) prepared from chitosan gel can be used in the detection of anions like F^−^ [96].

Wang et al. have developed a biocompatible PEG-chitosan@CDs hybrid nanogels by integrating nonlinear poly(ethylene glycol) (PEG), chitosan, and graphitic carbon dots (CDs) [36]. They have employed the developed hybrid nanogel in two-photon fluorescence (TPF) bioimaging, pH and near-infrared (NIR) light dual-responsive drug release, and synergistic therapy.

Chowdhury and co-workers [97] have reported a method of preparation of stable, soft but tough chitosan–carbon dots nanocomposite hydrogel films which they claim that the carbon dots synthesized from tea by green approach improved the strength, thermostability and wettability of chitosan hydrogel films. They have not used the hydrogel for any purpose like bioimaging, separation of heavy ions but claim their findings on the basis of result from UV/Vis spectroscopy, X-ray diffraction (XRD), FTIR, SEM, fluorescent microscopy, thermogravimetric analysis (TGA) and contact angle analysis.

Zhou et al. have shown that light-emitting polymer nanocomposites with excellent optical performance can be easily fabricated by the incorporation of amphibious CDs into the polymer matrix of peach gum polysaccharide (PGP) [45]. They have observed that the addition of CDs favored strong PL properties to polymer film.

### 4.2. CDs-Molecularly Imprinting Polymers (MIP) Complex

Molecular imprinting is one promising application to mimic nature and its principle is to prepare substrate-selective recognition sites in a matrix with a molecular template in a casting procedure [98,99,100]. The interesting scope of molecularly imprinting polymers has been elucidated in a review paper [101]. Several works are ongoing in the field where CDs are embedded in MIP to broaden the scope of the MIP. CDs embedded in molecularly imprinted polymer have been used in the recognition of sterigmatocystin, a fungal secondary metabolite produced by many different *Aspergillus* species, in grains [102]. In this study, CDs were directly encapsulated in a silica MIP in a one-pot reaction and it was found that PL properties of the CDs were not restricted by the silica-based MIP layer. They have suggested the no restriction phenomenon was due to optically transparent and chemically inert nature of silica but they have not discussed about the size fluctuations due to the fabrication which directly affects the PL. Furthermore, they have shown one method of detection of sterigmatocystin on the basis of PL quenching method because sterigmatocystin quenches the PL of CDs embedded in MIP.

A thermo-sensitive receptor CDs/SiO_2_/MIP was fabricated based on CDs and epitope approach, which they claim as the first reported approach [103]. In this research work, they have observed that the CDs/SiO_2_/MIP with double templates exhibited higher imprinting effect. The direct application of CDs/SiO_2_/MIP was subjected in the detection of target protein in the urine. Zhou and his co-workers have discussed an eco-friendly molecularly imprinted fluorescence composite material based on carbon dots fabricated via sol–gel polymerization for selective fluorescence detection of 4-nitrophenol (4-NP) of Yangtze River water samples [38]. According to their research work, the relative fluorescence intensity (*F*_0_/*F*) of MIP/CDs showed a good linearity with 4-NP concentrations in the linear range of 0.2–50 μmol·L^−1^ with a detection limit (3σ/*k*) of 0.06 μmol·L^−1^ having the correlation coefficient, imprinting factor as 0.9978 and 2.76 respectively.

Zuo et al. have reported nicotinic acid (NA) fluorescent optosensing based on molecular imprint technique [39]. In their research work, NA as template molecules were mixed with silane groups coated with CDs. The SiNPs were fabricated by sol-gel method by taking ethanol as a cross-linker and the recognition specificity of SiNPs along with the effect of NA on PL intensity was studied. The linear regression analysis helped them to determine the relationship between NA concentration and PL response which ultimately led the SiNPs to act as fluorescent probe for the detection of NA in several biological samples.

### 4.3. CDs Conjugated in Polymer Composite Films

The development of efficient, low-cost, and environmentally-friendly light emitters have gradually drawn intense attention due to their wide application in the lighting industry and photonics. In recent years, due to the high quantum efficiency, CDs were increasingly conjugated in polymer composite films to make white light emitters [45,104,105].

Since 2011, Wang and his coworkers initiated the first white light-emitting device originating from single CDs components [106]. Chen et al. have reported a white-light-emitting polymer composite films were synthesized by conjugating CDs and lanthanide complexes such as Eu(DBM)_3_ and Tb(DBM)_3_ (DBM: dibenzoylmethide) into a poly(methyl methacrylate) (PMMA) matrix [41]. The synthesized CDs emitted blue PL while the lanthanide complexes exhibited red and green PL. By adjusting the molar ratio of CDs and lanthanide complexes in the PMMA matrix, the high transparent polymer composite films could emit pure white PL.

**Figure 11:**
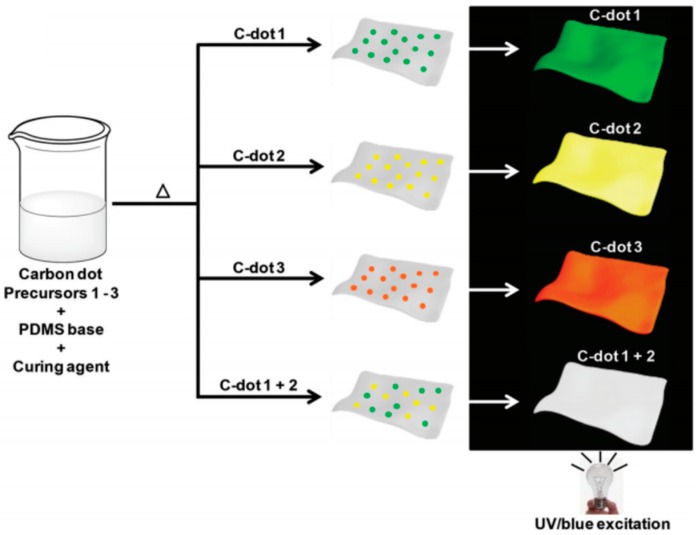
Phtoluminescent C-dot/PDMS films. Scheme describing the preparation of the mixed films. Distinct-colored C-dots embedded in the films were prepared by using different precursors. C-dot 1–3 indicates the carbon precursor is 6-O-(O-O′-Di-lauroyl-tartaryl)-d-glucose, 6-O-(O-O′-Di-lauroyl-tartaryl)-l-ascorbic acid, and Vitamin B1 + oleic acid, respectively. Figure adapted from Ref. [40] with permissions from the publishers.

Bhunia and her coworkers reported the fabrication of transparent films which exhibited tunable light emission through one-pot synthesis of polymer matrixes with embedded CDs (C-dot) assembled simultaneously with the polymer matrix [40]. Interestingly, the films exhibit different distinct PL color based on different carbon precursors. Besides, by mixing carbon precursors, the CDs film exhibited different PL color including white color (Figure 11).

Zeng and Yan have prepared luminescent cellulose film by combination of cellulose and CDs in aqueous solution [30]. In doing so, they fabricated a biocompatible transparent material by green process with potential applications in optical devices, biomedicine and environmentally benign packaging. They studied the mechanical performance of the regenerated cellulose and composite cellulose/CDs films by stress-strain (σ–ε) curves. They reported that tensile strength and elongation at breaking of the pure cellulose film were 46.9 MPa and 4.1%, respectively. Furthermore, the elastic modulus and tensile strength were found to be 344 and 58.5 MPa, respectively. From these data, they confirmed that the composite was softer than that of the regenerated cellulose film.

Besides, the luminescent C-dots have been covalently attached in aqueous media to cellulose nanofibrils via *N*-(3Dimethylaminopropyl)-*N*′-ethylcarbodiimide hydrochloride/*N*-hydroxysuccinimide (EDC/NHS) coupling by Junka et al. [107]. In this study, they found that C-dots do not aggregate during drying while embedded in the carboxymethylated-cellulose nanofibrils (CM-CNF) matrix and there was increment in thermal stability due to the attachments of C-dots. Transparent, smooth, and fluorescent nanopaper was produced from CNF-C-dots. However, they have not demonstrated the potential of C-dots modified by CM-CNF in anticounterfeit and biosensing applications.

**Figure 12:**
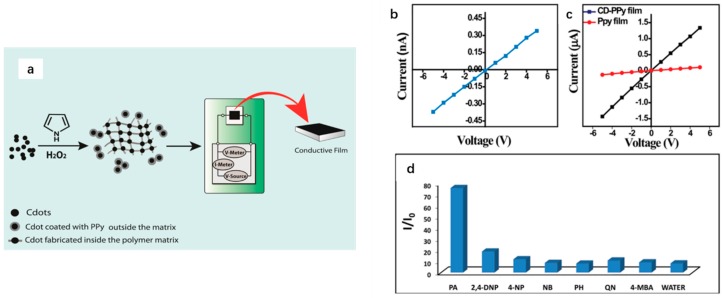
(**a**) Schematic representation of the synthesis of CDs-PPy composite and the primary set up for the conductivity experiment of the picric acid. Plot of *I*–*V* characteristics of: (**b**) CDs film; and (**c**) CD-PPy film and PPy film. (**d**) The ratios of current flowing through the composite film after adding 2.0 μL 1.0 mM aqueous solution of different analytes to that of the film only. Here PA = picric acid, 2,4-DNP = 2,4-dinitrophenol, 4-NP = 4-nitrophenol, NB = nitrobenzene, PH = phenol, QN = 1,4-benzoquinone, 4-MBA = 4-methoxybenzoic acid. The measurements were made at +5 V. Figure adapted from Ref. [108] with permissions from the publishers.

Except high PL efficiency, the high electrical conductivity is also attractive for CDs to work as a sensitive sensor. In 2016, Pal et al. synthesized a conducting nanocomposite consisting of CDs and polypyrrole (PPy) (a p-type semiconductor) and it is the first report on the sensing work based on conductivity of CDs (Figure 12a) [108]. The conductivity of the CDs film (Figure 12b) was explained by the presence of sp^2^ C=C bonds and the introduction of CDs into the polymer composite contribute to higher conductivity of the composite nanomaterial than PPy (Figure 12c), which was due to the enhancement of enrichment of electron acceptors of CDs. As is illustrated in Figure 12d, the response of the composite film was very strong toward picric acid which is widely used to produce explosives and considered as a pollutant to contaminate groundwater and soil. Therefore, the synthesized CDs films can work as a great biosensor to selectively and sensitively detect the presence of picric acid in aqueous phase as well as soil.

## 5. Conclusions

In this review, we tried to highlight and critically analyze the ongoing trends in CDs linked to polymers, focusing on the key role played by polymers in the formation of CDs and the fabrication of CDs–polymer composite with their actual applications. As a good template of CDs, polymers themselves own repeated monomer units which is beneficial for the analysis of the CDs structure. Besides, most CDs formation needs to experience polymerization while different monomers with different molar ration will vary the PL properties. Therefore, the selection of proper monomers is important. In addition, CDs passivation technique has been applied to improve the PL of CDs, which involves polymers such as PEG series compounds and PEI which render the PL of CDs. In terms of application, different kinds of composites ranging from molecularly imprinted polymers to drug free hybrid nanogels have promising characteristics in fields from bioimaging to drug delivery. The hydrogels and polymer composites revealed excellent features such as convenient synthesis, low cost, large surface area (relative to volume), enhanced PL, selectivity, stability, pH dependent drug release, etc.

## Figures and Tables

**Table 1:** CDs obtained from CA coupled with amine compounds and their PL properties.

Reaction Condition	Carbon Source	Passivation Agent	UV/Vis (nm)	PL Max Em (nm)	QY (%)	Application	Ref.
200 °C	CA	EDA	238 350	445	69.3	Bioimaging	[56]
200 °C	CA	EDA	344	445	80	Printing Fe^3+^ detection	[61]
120 °C	CA	EDA	340	440	21.8	N/A	[62]
150 °C	CA	LPEI	249 355	445	37.4	N/A	[58]
250 °C	CA	Tris	238 330	410	52	Biosenser Fe^3+^ detection	[63]
700 W	CA	Urea	334 408	420	12	ClO^−^ and ONOO^−^ detection	[64]
200 °C	CA	ammonia	335	550	36	Solar cell	[65]
700 W	CA	tryptophan	280	450	20.6	Bioimaging Nanocarrier	[66]
240 °C	CA	melamine	270 320	390	42	Fe^3+^ detection	[67]
180 °C	CA	1,10-phenanthroline	271 310	440	10	Fe^3+^ and Fe^2+^ detection	[68]
500 W	CA	tetraoctylammonium	280 330	375	11	Bioimaging	[69]
180 °C	CA	PEI	247 357	475	24.3	Biosenser Bioimaging	[70]
